# A simple method for RNA isolation from various tissues of the tree *Neolamarckia cadamba*

**DOI:** 10.1080/13102818.2014.981086

**Published:** 2014-11-21

**Authors:** Kunxi Ouyang, Juncheng Li, Hao Huang, Qingmin Que, Pei Li, Xiaoyang Chen

**Affiliations:** ^a^ State Key Laboratory for Conservation and Utilization of Subtropical Agro-bioresources, Guangdong Key Laboratory for Innovative Development and Utilization of Forest Plant Germplasm, South China Agricultural University, Guangzhou, Guangdong, P.R. China; ^b^ Guangxi Botanical Garden of Medicinal Plants, Nanning, Guangxi, P.R. China; ^c^ Key Laboratory for Genetics and Breeding of Forest Trees and Ornamental Plants, Ministry of Education, National Engineering Laboratory for Forest Tree Breeding, Beijing Forestry University, Beijing, P.R. China

**Keywords:** *Neolamarckia cadamba*, RNA extraction, CTAB, spermidine, recalcitrant plant tissues

## Abstract

Plant tissues contain abundant polysaccharides, phenolic compounds and other metabolites, which makes it difficult to isolate high-quality RNA from them. In addition, *Neolamarckia cadamba* contains large quantities of other components, particularly RNA-binding alkaloids, which makes the isolation even more challenging. Here, we describe a concise and efficient RNA isolation method that combines the cetyltrimethyl ammonium bromide (CTAB) and Plant RNA Kit (Omega) protocols. Gel electrophoresis showed that RNA extracted from all tissues, using this protocol, was of good integrity and without DNA contamination. Furthermore, the isolated RNA was of high purity, with an *A*_260_/*A*_280_ ratio of 2.1 and an *A*_260_/*A*_230_ ratio of >2.0. The isolated RNA was also suitable for downstream applications, such as reverse transcription-polymerase chain reaction (RT-PCR) and quantitative RT-PCR (RT-qPCR). The RNA isolation method was also efficient for recalcitrant plant tissues.

## Abbreviations

cDNA: complementary DNA
CTAB: cetyltrimethyl ammonium bromide
DEPC: diethyl pyrocarbonate
EDTA: ethylenediaminetetraacetic acid
PVP: polyvinylpyrrolidone
RNase: ribonuclease
rRNA: ribosomal RNA
RT-PCR: reverse transcription-polymerase chain reaction

## Introduction

*Neolamarckia cadamba* is a synonym for *Anthocephalus chinensis* and belongs to the family Rubiaceae. This is a fast-growing evergreen broadleaf tree species distributed in tropical and southern Asia.[1] It was universally accepted as ‘a miraculous tree’ at the World Forestry Congress in 1972, because of its rapid growth. This species also has significant development and utilization value in South China. To date, research has been focused on the medicinal compounds present in *N. cadamba*, particularly alkaloids,[2–5] but few studies of its molecular biology, based on isolation of high-quality RNA, have been performed.

The isolation of high-quality RNA lacking polysaccharides, proteins, phenolic compounds, genomic DNA or secondary metabolites is crucial for the multiple techniques used to investigate gene expression patterns and functions during plant growth and development. Such techniques are reverse transcription-polymerase chain reaction (RT-PCR), quantitative RT-PCR (RT-qPCR), complementary DNA (cDNA) library construction, Northern blotting and RNA sequencing. Phenolic compounds are oxidized to form quinones, which bind irreversibly to nucleic acids and proteins.[6] Polysaccharides can co-precipitate and degrade RNA, which renders RNA unsuitable for downstream applications. In addition, *N. cadamba* contains abundant alkaloids that bind RNA, increasing the difficulty of isolation.[7–9] Currently commercially available kits, such as those manufactured by Takara, Promega, Omega and Qiagen, did not enable extraction of RNA from all *N. cadamba* tissues (data not shown), may be because the efficiency of the spin columns used in these kits decreases significantly in the presence of alkaloid compounds.[10] Ouyang et al. [1] isolated RNA from four young tissues to clone genes, using an RNeasy Plant Mini Kit (Qiagen), but the concentration and/or quantity of the extracted RNA were not sufficient for cDNA library construction, Northern blotting or RNA-sequencing. The only exception was the RNA extracted from the cambium region of the tree. Тo our knowledge, there is no report on total RNA extraction from the other tissues of *N. cadamba*.

RNA has been successfully extracted from various plant species, rich in phenolic compounds and polysaccharides, using the cetyltrimethyl ammonium bromide (CTAB) protocol,[10–14] but this method is time consuming. A combination of the CTAB-based RNA extraction method and a commercial plant RNA extraction kit has been successfully utilized.[10,15] However, RNA extracted from some *N. cadamba* tissues, according to these protocols, smeared severely (data not shown). Here, we combined a modified CTAB lysis buffer and purification on RNA mini columns (Omega), which not only reduced the required time but also yielded large quantities of high-quality total RNA.

## Materials and methods

### Plant materials

Four young tissues, buds, leaves, cambium scrapings and roots, were collected as described previously.[1] Flowers at full bloom, fruits at flower fall, five-centimeters-high seedlings and young shoot segments without bark below the buds were also collected. The other recalcitrant plant tissues used in this study are listed in Table 2. After collection, all plant materials were immediately frozen in liquid nitrogen and stored at −80 °C until needed.

**Table 1.** Quality of RNA extracted from seven tissues.

	NanoDrop 1000^a^			2100 Bioanalyzer^b^	
Plant tissue	*A*_260_/*A*_280_	*A*_260_/*A*_230_	ng/μL	RIN^c^	rRNA ratio (28S/18S)
Bud	2.14 ± 0.04	2.12 ± 0.01	1014 ± 167	9.4	2.2
Leaf	2.16 ± 0.02	2.21 ± 0.21	2238 ± 430	8.7	1.5
Cambium region	2.13 ± 0.01	2.12 ± 0.12	1119 ± 214	9.5	1.9
Root	2.14 ± 0.10	2.13 ± 0.14	945 ± 231	8.9	2.2
Shoot segment	2.14 ± 0.03	2.17 ± 0.09	723 ± 35	9.6	2.0
Flower	2.14 ± 0.01	2.06 ± 0.23	260 ± 7	9.2	2.4
Fruit	2.19 ± 0.00	2.25 ± 0.05	1044 ± 100	9.1	2.4
Seedling	2.17 ± 0.01	2.16 ± 0.17	1331 ± 146	9.1	2.7

^a^Results represent the means ± standard deviation of three samples.

^b^The results of one biological replicate are shown.

^c^RIN – RNA integrity number.

**Table 2.** Purity and yield of total RNA extracted from recalcitrant plant tissues.

Plant species	Tissues	*A*_260_/*A*_280_	*A*_260_/*A*_230_	Concentration (ng/μL)
*Camellia sinensis* L. (tea) [31]	Bud	2.12	1.98	3032
	Young leaf	2.2	2.05	3519
	Fully expanded leaf	2.18	1.93	736
				
*Eriobotrya japonica* Lindl. (loquat) [32]	Terminal bud	2.19	1.95	670
	Young leaf	2.16	2.04	735
	Adult leaf	2.15	2.18	637
				
*Pinus taeda* L. (loblolly pine) [33]	Young needle	1.86	1.94	296
	Adult needle	2.01	1.90	273
				
*Litchi chinensis* Sonn. (lychee) [34]	Young leaf	2.19	2.05	1924*
	Adult leaf	2.18	2.18	1180
				
*Rosa chinensis* (rose) [32]	Young petal	1.82	1.97	445
	Adult petal	1.80	1.95	388
				
*Taxus media* (taxus) [35]	Young leaf	2.13	2.14	3150*
	Adult leaf	2.04	2.18	2873
				
*Ginkgo biloba* L. (ginkgo) [35]	Young leaf	2.01	1.93	447
	Adult leaf	2.16	2.12	460
				
*Opuntia ficus-indica* L. (cactus) [36]	Cladode	2.05	1.95	255*
				
*Aloe barbadensis* Mill. (curacao aloe) [36]	Leaf	2.18	2.21	277*

Note: *The yield given by the simple method was higher than that in the corresponding reference described by *p* < 0.05.

### RNA extraction

Frozen tissue was ground to a fine powder in liquid nitrogen, using a mortar and pestle. Then, 100 mg samples of the powder were transferred into individual 1.5 mL RNase-free tubes containing 600 μL of prewarmed extraction buffer at 60 °C. The extraction buffer contained the following: 2% CTAB, 2% polyvinylpyrrolidone (PVP) K-40, 100 mmol/L Tris–HCl (pH 8.0), 25 mmol/L ethylenediaminetetraacetic acid (EDTA; pH 8.0), 2.0 mol/L NaCl, 2 g/L spermidine and 2% β-mercaptoethanol (added immediately before use). Lysis buffer without spermidine or β-mercaptoethanol was treated with 0.1% diethyl pyrocarbonate (DEPC) and autoclaved. 2 g/L spermidine was added and the mixture was stored at room temperature. The extracts were mixed by vortexing and incubated at 60 °C in a water bath for 10 min with vigorous shaking for several times. An equal volume of chloroform/isoamyl alcohol (24:1) was added to the homogenate and was mixed completely by vortexing. The mixture was centrifuged at 12,000 r/min for 10 min at 4 °C, except for bud samples, which were centrifuged for 20 min. The supernatant was transferred to a new tube and the above step was repeated. The supernatant was then transferred to a new tube containing an equal volume of RB buffer from the Plant RNA Kit (Omega); an equal volume of ethanol was then added to each tube. The mixture was blended by inverting the tube and was filtered through an RNA mini column (Omega), according to the manufacturer's protocol. The final steps were according to this protocol and RNA was eluted with 40 μL of DEPC H_2_O.

### RNA analysis

The RNA concentration was determined by measuring the absorbance at 230, 260 and 280 nm, using a spectrophotometer (NanoDrop 1000, USA). The purity of the RNA was estimated by calculating the *A*_260_/*A*_280_ and *A*_260_/*A*_230_ ratios to evaluate the levels of protein and polysaccharide/phenolic compound contamination, respectively. The integrity of total RNA was verified by resolving a ∼1 μg RNA sample on a 1.2% (w/v) formaldehyde denaturing agarose gel with DNA marker DL2000 as control and 6× loading buffer (30 mmol/L EDTA, 36% glycerol, 0.035% xylene cyanol and 0.05% bromophenol blue) as staining buffer. Further analysis was done in an Agilent 2100 Bioanalyzer to calculate RIN (RNA integrity number) values.

### cDNA synthesis and real-time RT-PCR

Total RNA (0.5 μg) was reverse-transcribed to first-strand cDNA, according to the manufacturer's instructions in the PrimeScript™ RT Master Mix kit (Takara, Japan). According to Xiao et al.,[16] for further confirmation of the quality of total RNA extracted by this protocol, serial dilutions (1:1, 1:5, 1:25, 1:125 and 1:625) of the single-stranded cDNA were subjected to quantitative real-time RT-PCR. Real-time PCR was performed following the standard SYBR Premix Ex Taq™ kit (Takara, Japan) protocol, using a final volume of 20 μL which includes 2 μL of reverse-transcribed cDNA and 2 μL of 5 μmol/L forward and reverse primers. Thermocycling conditions were as follows: an initial denaturation at 95 °C for 30 s, followed by 40 cycles of 95 °C for 5 s, 58 °C annealing for 30 s and 72 °C extension for 15 s and an infinite hold at 10 °C. The specificity of the PCR amplification procedures was checked using a heat dissociation protocol (from 65 to 95 °C) after the final PCR cycle and was examined by electrophoresis in 2% agarose gel. The sequences of forward and reverse primers for the cyclophilin gene (JX902587) were 5′-GACAGGAGGAGAATCTATCTATGG-3′ and 5′-AACCTGCCCAAACACCACAT-3′, respectively.

## Results and discussion

### RNA analysis

The RNA isolation protocols of currently commercially available kits and reagents are simple, rapid, non-toxic and give good yields of high-quality RNA from suitable plant tissues. However, good yields cannot be obtained, or RNA cannot be extracted from recalcitrant plant tissues, using these kits or reagents. Several commercially available kits and reagents, such as Promega, Omega and TRIzol, failed to yield good-quality RNA isolated from leaves, buds, flowers and fruits of *N. cadamba*. Other methods were also ineffective.[15–21]

The CTAB protocol is used widely for extraction of RNA from plant tissues but is tedious and time consuming. In the present study, we introduced several changes to the original CTAB protocol with inclusion of OMEGA kit-based steps; this method facilitated the isolation of large quantities of high-quality RNA from various *N. cadamba* tissues in 2 h.

**Figure 1.**
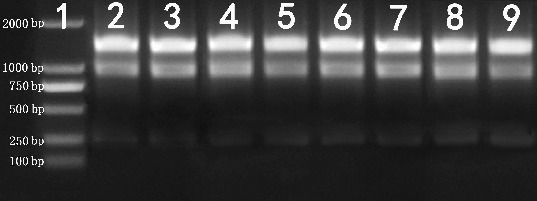
Agarose gel electrophoresis of total RNA (1 μg) extracted from *N. cadamba* tissues. Note: Lane 1 – DNA molecular weight marker; Lane 2 – bud; Lane 3 – leaf; Lane 4 – cambium region; Lane 5 – root; Lane 6 – young shoot segment; Lane 7 – flower; Lane 8 – fruit; Lane 9 – seedling.

The extraction protocol described in this paper gave a high amount of good-quality total RNA without degradation from eight tissues of *N. cadamba*. Pure RNA has an *A*_260_/*A*_280_ ratio of 1.8–2.2 and an *A*_260_/*A*_230_ ratio of >2.0.[22] In all samples, the *A*_260_/*A*_230_ ratio ranged from 2.06 to 2.25, indicating that the RNA was of high purity and free of polyphenol and polysaccharide contamination. Similarly, the *A*_260_/*A*_280_ ratio ranged from 2.13 to 2.19, which also indicated lack of protein contamination (Table 1). The assessment of RNA integrity showed distinct 28S and 18S ribosomal RNA (rRNA) bands without smearing for all RNA samples that we tested (Table 1, Figure 1 and Figure S1 in the online Supplementary Appendix), indicating that the RNA samples were not degraded.

In RNA extraction protocols, the strong reductant β-mercaptoethanol is conventionally used at 2% (v/v) to denature RNases and other contaminating proteins released during tissue disruption and homogenization.[11–13,23–25] By using Gasic's RNA extraction buffer with 2% β-mercaptoethanol,[26] RNA was successfully extracted from the cambium region, root, young shoot segment and seedling, but the RNA extracted from the four other tissues (flower, fruit, leaf and bud) smeared severely after elution from the silicon column. Even with 5% β-mercaptoethanol, RNase activity was not inhibited, as evidenced by the severe smearing of RNA (data not shown). Spermidine, in Gasic's RNA extraction buffer, is an RNase inhibitor.[26–30] However, RNase activity was not inhibited by 0.5 g/L of spermidine, resulting in smearing of RNA extracted from buds, leaves, flowers and fruits (data not shown). The RNA extracted from these tissues also smeared severely even with 1 g/L of spermidine, as in Sangha's protocol,[15] because RNase activity was not inhibited. However, 2 g/L of spermidine in the RNA extraction buffer resulted in isolation of high-integrity RNA that exhibited distinct bands for 28S and 18S rRNA (Figure 1 and Figure S1 in the online Supplementary Appendix). Thus, the RNase activity in various *N. cadamba* tissues was effectively inhibited by inclusion of 2 g/L of spermidine in the RNA extraction buffer.

**Figure 2.**
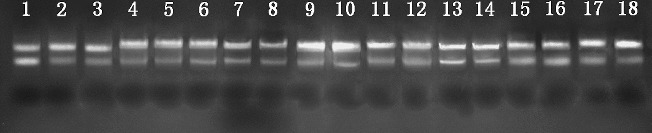
Agarose gel electrophoresis of total RNA extracted from 18 recalcitrant plant tissues by the modified protocol. Note: Lanes 1–3: tea (bud, young leaf and fully expanded leaf, respectively); Lanes 4–6: loquat (terminal bud, young leaf and adult leaf, respectively); Lanes 7 and 8: loblolly pine (young needle and adult needle); Lanes 9 and 10: lychee (young leaf and adult leaf); Lanes 11 and 12: rose (young petal and adult petal); Lanes 13 and 14: taxus (young leaf and adult leaf); Lanes 15 and 16: ginkgo (young leaf and adult leaf); Lane 17: cactus cladode; Lane 18: curacao aloe leaf.

To inspect whether the RNA extraction protocol is also suitable for total RNA isolation from a wider range of recalcitrant plant tissues, 18 tissue samples from nine plant species [31–36] were taken and immersed in liquid nitrogen for subsequent RNA isolation. The protocol, when applied to these recalcitrant plant tissues, not only gave high-quality total RNA with *A*_260_/*A*_280_ ≥ 1.8 (Table 2) and two sharp and well-resolved rRNA bands (Figure 2), but also saved time and gave a higher yield than previous protocols in some samples, such as lychee,[34] taxus,[35] cactus and curacao aloe.[36]

### Quantitative real-time RT-PCR

**Figure 3.**
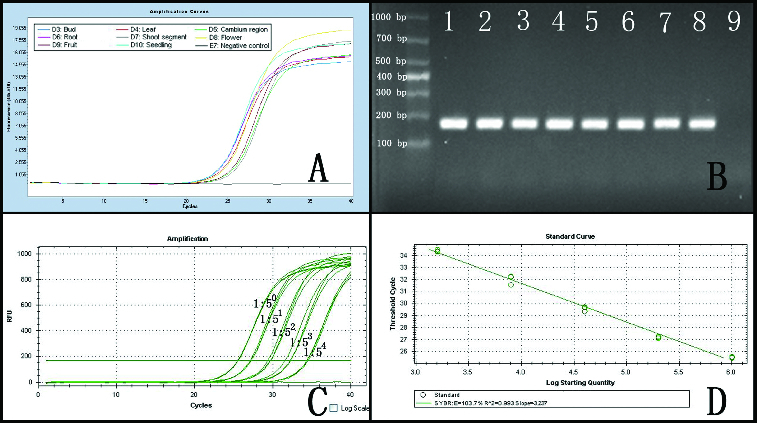
Real-time RT-PCR amplification curve of the cyclophilin gene. Note: (a) Amplification of the cyclophilin gene from RNA extracted from eight tissues. (b) Electrophoresis of amplicons from (a); Lane 1 – bud; Lane 2 – leaf; Lane 3 – cambium region; Lane 4 – root; Lane 5 – young shoot segment; Lane 6 – flower; Lane 7 – fruit; Lane 8 – seedling; Lane 9 – negative control. (c) Real-time RT-PCR of the cyclophilin gene with serial dilutions of cDNA from the cambium region (1:1, 1:5, 1:25, 1:125 and 1:625). (d) Standard curve generated from data in (c).

The RNA extracted from *N. cadamba* was amplified by quantitative RT-PCR, yielding smaller amplicons (181 bp), as shown in Figure 3. The cyclophilin gene was amplified from the eight cDNA samples, using real-time RT-PCR (Figure 3(a)). Electrophoresis of amplicons indicated that the primer pair used to amplify the cyclophilin gene fragment exhibited high specificity (Figure 3(b)). The amplification curves demonstrated that these smaller mRNA sequences were largely intact in the RNA samples and that the cyclophilin gene was present in all tissues in moderate abundance. A dilution series (1:1, 1:5, 1:25, 1:125, 1:625) of single-strand cDNA from the cambium region RNA extracted by this protocol showed normal amplification curves; the amplification efficiency was 103% (Figure 3(c) and 3(d) and Figure S2 in the online Supplementary Appendix). The other tissue cDNA samples yielded similar results, indicating that the quality of the extracted RNA met the basic experimental needs.

## Conclusions

Our results demonstrated that, using a modified CTAB protocol, a large quantity of high-quality total RNA can be isolated from various *N. cadamba* tissues. This type of RNA cannot be isolated by other methods due to the presence of abundant alkaloids, high RNase activity and polysaccharides, polyphenols and secondary metabolites. The method is also cost effective, time saving and universal. This simple protocol can be used as an alternative method for RNA isolation from recalcitrant plant tissues.

## Supplemental data

Supplemental data for this article can be accessed at http://dx.doi.org/10.1080/13102818.2014.981086.
